# An optimised whole mount *in situ* hybridisation protocol for the mollusc *Lymnaea stagnalis*

**DOI:** 10.1186/s12861-015-0068-7

**Published:** 2015-03-28

**Authors:** Jennifer Hohagen, Ines Herlitze, Daniel John Jackson

**Affiliations:** Department of Geobiology, Geosciences Centre, Georg-August University of Göttingen, Goldschmidtstrasse 3, 37077 Göttingen, Germany

**Keywords:** Whole mount *in situ* hybridisation, Mollusc, *Lymnaea stagnalis*, Gene expression, Evolution, Development

## Abstract

**Background:**

The ability to visualise the expression of individual genes *in situ* is an invaluable tool for developmental and evolutionary biologists; it allows for the characterisation of gene function, gene regulation and through inter-specific comparisons, the evolutionary history of unique morphological features. For well-established model organisms (e.g., flies, worms, sea urchins) this technique has been optimised to an extent where it can be automated for high-throughput analyses. While the overall concept of *in situ* hybridisation is simple (hybridise a single-stranded, labelled nucleic acid probe complementary to a target of interest, and then detect the label immunologically using colorimetric or fluorescent methods), there are many parameters in the technique that can significantly affect the final result. Furthermore, due to variation in the biochemical and biophysical properties of different cells and tissues, an *in situ* technique optimised for one species is often not suitable for another, and often varies depending on the ontogenetic stage within a species.

**Results:**

Using a variety of pre-hybridisation treatments we have identified a set of treatments that greatly increases both whole mount *in situ* hybridisation (WMISH) signal intensity and consistency while maintaining morphological integrity for early larval stages of *Lymnaea stagnalis*. These treatments function well for a set of genes with presumably significantly different levels of expression (*beta tubulin*, *engrailed* and *COE*) and for colorimetric as well as fluorescent WMISH. We also identify a tissue-specific background stain in the larval shell field of *L. stagnalis* and a treatment, which eliminates this signal.

**Conclusions:**

This method that we present here will be of value to investigators employing *L. stagnalis* as a model for a variety of research themes (e.g. evolutionary biology, developmental biology, neurobiology, ecotoxicology), and brings a valuable tool to a species in a much understudied clade of animals collectively known as the Spiralia.

**Electronic supplementary material:**

The online version of this article (doi:10.1186/s12861-015-0068-7) contains supplementary material, which is available to authorized users.

## Background

Analysing how spatial and temporal developmental gene expression profiles evolve is a powerful strategy for understanding how morphological diversity can be generated. The most commonly employed technique for the study of spatial gene expression in a given tissue or developmental stage is *in situ* hybridisation (ISH), often applied to whole embryos or larvae as whole mount *in situ* hybridisation (WMISH). WMISH provides information about the timing and localisation of a gene’s expression in a developing embryo or larva, and can be used to characterise and identify cell types, tissues or organs within the whole organism and to make inferences about their function and evolutionary history [1-3]. Unfortunately, the technique can be challenging, especially when applied to an organism for which there is little knowledge regarding the multifarious conditions that optimise the balance between WMISH signal intensity and the preservation of morphological integrity, two often conflicting requirements. WMISH experiments on embryos can be further challenged by changes in the biochemical and biophysical properties of the developing tissues during ontogenesis. Thus, the procedure often needs to be adapted for distinct developmental stages within a species.

**Figure 1:**
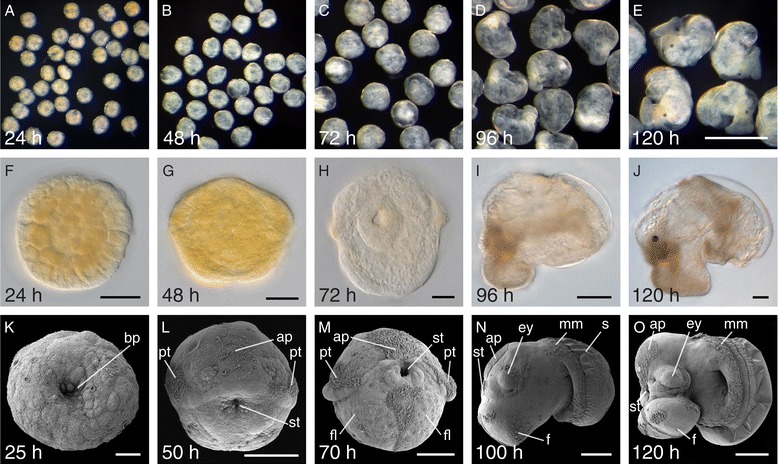
**Overview of the early larval development of** ***L. stagnalis*** **.** During the first five days of development, embryos of *L. stagnalis* undergo drastic changes in size (**A-E**, images are to the same scale shown in **E**), tissue composition **(F-J)** and form all main larval structures **(K-O)**. Indicated are the positions of the apical plate (ap), the eye (ey), the foot lobe (fl) or foot (f), the developing mantle margin (mm), the prototroch (pt), the shell (s) and the blastopore (bp) or stomodaeum (st). All ages are indicated in hours post first cleavage (h). **F-H** and **K-M** are ventral views with scale bars representing 50 μm. **I**, **J**, **N** and **O** are lateral views with scale bars of 100 μm. The scale bar in panel **E** is 500 μm. Panels **I**, **J**, **N** and **O** are reflected about the vertical axis for consistency of presentation.

From an evo-devo perspective, the pulmonate freshwater gastropod *Lymnaea stagnalis* (Linnaeus, 1758) is a representative of a significantly understudied group of animals, the Spiralia/Lophotrochozoa. Primarily due to its availability and ease of culture, *L. stagnalis* was once a much used model for studying molluscan development [4-6], and is currently employed as a model for studies focused on various biological processes including the establishment of chirality [7], the evolution of shell formation [8] and ecologically regulated development [9]. However, *L. stagnalis* possesses certain traits that represent technical challenges to WMISH. First, *L. stagnalis* embryos develop individually within egg capsules filled with a fluid that serves a nutritive function and is uptaken by pinocytosis during development [10-12]. This viscous intra-capsular fluid, which consists of a complex mixture of ions, polysaccharides, proteoglycans and other polymers [13], can be seen to stick to the embryo following decapsulation, and likely interferes with any WMISH procedure. Second, from 52 hours post first cleavage onwards the first insoluble material associated with shell formation is secreted [8]. This material non-specifically binds some nucleic acid probes and generates a characteristic background signal. This phenomena is not restricted to *L. stagnalis* but can be observed in larvae of other gastropods (our unpublished data), bivalves, scaphopods and polyplacophoran molluscs (pers. comm. Tim Wollesen). Finally, *L. stagnalis* embryos and larvae undergo significant morphometric and biophysical changes in the characteristics of their tissues during the first days of development (Figure 1). Previously described WMISH protocols for larvae of *L. stagnalis* produced WMISH signals with low signal to noise ratios, making some previously reported gene expression patterns difficult to interpret [14-16].

In order to achieve consistent WMISH signals in *L. stagnalis* larvae with maximum signal to noise ratios, we have systematically compared the influence of a variety of chemical and enzymatic pre-hybridisation treatments previously reported to address each of these challenges. We first evaluated the effect of the mucolytic agent N-acetyl-L-cysteine (NAC) in order to assess the possibly negative influence of the intra-capsular fluid on WMISH in *L. stagnalis*. A treatment with NAC has been shown to improve WMISH signal intensity in the platyhelminth flatworm *Schmidtea mediterranea,* presumably by degrading the mucosal layer surrounding the animal and thereby increasing accessibility of the probe to the tissue [17]. WMISH signal quality was also improved in *S. mediterranea* through the use of the reducing agent dithiothreitol (DTT) and the detergents sodium dodecyl sulfate (SDS) and NP-40, a treatment referred to by Pearson *et al*. as ‘reduction’ [17]. An alternative permeabilising treatment solely utilising SDS is commonly employed in WMISH protocols for a variety of animals such as the platyhelminth *S. mediterranea* [18] or the arthropod *Parhyale hawaiensis* [19,20]. Here we assess the impact of different combinations of these and other standard WMISH treatments (storage, enzymatic permeabilisation by Proteinase K (Pro-K), acetylation) on the strength and consistency of the WMISH signal across early developmental stages of *L. stagnalis*. We also systematically evaluated the effects of the Alkaline Phosphatase (AP) -conjugated anti-DIG antibody concentration, the composition of the colour detection solution and different probe preparation approaches. We have performed these experiments with a selection of three genes, which can be reasonably assumed to have different levels of expression: *beta tubulin,* and the transcription factors *engrailed* and *COE* (*collier/olfactory-1/early B cell factor*). We also demonstrate the presence of a tissue-specific background stain, which can be abolished by treatment with triethanolamine (TEA) and acetic anhydride (AA). The optimised WMISH method we present here will allow for future molecular studies to be performed on a wide range of developmental processes within *L. stagnalis*.

## Methods

A detailed list of all solutions used can be found at the end of this section. If not otherwise indicated, all steps were carried out at room temperature.

### Cultivation of adult L. *stagnalis* and preparation of embryos

Laboratory cultures derived from adult *L. stagnalis* collected from the Northeimer Seenplatte, Germany, from a pond on the North campus of the University of Göttingen, Germany, and from Nottingham, U.K. and were kept in standard tap water at 25°C, under a 16:8 light dark regime and fed *ad libidum* with lettuce and a variety of other vegetables. Under this regime adult snails lay egg masses year round. Egg masses of diverse ages were collected and grouped into three developmental time windows: from one to two days post first cleavage (dpfc), from approximately two to three dpfc and from three to five dpfc. Individual egg capsules were freed from the surrounding jelly by rolling them over moist filter paper. Embryos were released from their egg capsules by manual dissection using forceps and mounted needles. In order to minimise experimental error, embryos for each experiment were pooled and processed up to a point when experiment-specific treatments were applied.

### NAC treatment

Freshly dissected embryos were immediately incubated in NAC solution. The duration and concentration of this treatment were age-dependent. Embryos ranging from two to three dpfc were treated for five minutes with 2.5% NAC, and samples between three and six dpfc were treated with 5% NAC twice for five minutes each. All samples were then immediately fixed for 30 minutes in 4% paraformaldehyde (PFA) in PBS.

### Fixation

All samples were transferred into freshly prepared 4% (w/v) PFA in 1X PBS and incubated for 30 minutes at room temperature. The fixative was removed by one wash for five minutes in 1X PBTw. Samples were then subjected to a treatment with SDS.

### SDS treatment

Following fixation, all samples were washed once in PBTw for five minutes and then incubated in 0.1% SDS, 0.5% SDS or 1% SDS in PBS for ten minutes at room temperature. Following the SDS treatment, samples were rinsed in PBTw and dehydrated through a graded ethanol (EtOH) series in PBTw; one wash in 33% (v/v) EtOH, one wash in 66% (v/v) EtOH and two washes in 100% EtOH, each wash lasting five to ten minutes. All samples were then stored at −20°C.

### Reduction

Following fixation and one five minutes wash in PBTw, embryos between two and three dpfc were treated with 0.1X reduction solution for ten minutes at room temperature. Embryos between three and five dpfc were incubated for ten minutes in preheated 1X reduction solution at 37°C. All samples were carefully inverted once during this time. We found all samples to be extremely fragile in this solution and should be handled with care. After removal of the reduction solution, all samples were briefly rinsed with PBTw before being dehydrated through a graded EtOH series; one wash in 50% (v/v) EtOH, two washes in 100% EtOH, each wash lasting five to ten minutes. All samples were then stored at −20°C. Note: this treatment replaces the SDS treatment described above.

### RNAse treatment in order to investigate the source of non-specific WMISH staining

NAC-treated samples were fixed and dehydrated as described above and stored at −20°C. Samples were then rehydrated through a graded EtOH series into PBTw and then incubated for 30 minutes at 37°C in 10 μg/ml and 100 μg/ml RNAse A (Sigma, #R5503) in 2X SSC. Samples were then washed five times in PBTw for 5 minutes each before proceeding with Proteinase K digestion.

### Protein digestion with Proteinase K (Pro-K)

Following fixation, dehydration, storage at −20°C and any additional treatments (NAC, reduction or SDS), samples were rehydrated through a graded EtOH series into PBTw. Embryos were then treated with an age-dependent concentration of Pro-K (Carl Roth, #7528) for ten minutes at room temperature. The regimes ultimately employed are the culmination of a more exhaustive series of trials using a greater range of Pro-K concentrations (0 to 50 μg/ml Pro-K). Embryos between one and two dpfc were incubated in concentrations of Pro-K ranging from 1–15 μg/ml, embryos between two and three dpfc in concentrations of Pro-K ranging from 5–20 μg/ml and older embryos (between three and five dpfc) were treated at concentrations between 5 μg/ml and 40 μg/ml. Pro-K activity was stopped by two five minutes washes in 2 mg/ml glycine. All samples were then briefly rinsed in PBTw.

### Triethanolamine + acetic anhydride (TEAAA) treatment

Samples were transferred into a 1% (v/v) solution of triethanolamine (TEA) in PBTw and incubated for five minutes. This step was then repeated. This solution was then replaced with a solution of 1% TEA + 0.3% (v/v) acetic anhydride (TEAAA) in PBTw. This step was repeated for some samples. All samples were then washed once with PBTw, postfixed for 15 to 20 minutes in 4% PFA in PBTw, and washed three times with PBTw before being transferred into an Intavis In situ-Pro robot for all subsequent hybridisation, antibody incubation and washing steps.

### Riboprobe synthesis

Primers designed to amplify fragments of *beta tubulin, engrailed* and *COE* were designed from 454 and Illumina RNASeq data (see Additional file 1 for all primer sequences). These PCR products were cloned into vectors containing T7 and SP6 promotor sites and verified by Sanger sequencing. These fragments were then amplified from plasmid DNA using M13 primers, and purified using the QIAGEN QIAquick Gel Extraction Kit. Antisense riboprobes were synthesised using Promega reagents in a 10 μl-reaction containing 1X reverse transcription buffer, 10 mM Dithiothreitol, 1X Digoxigenin RNA labeling Mix (Roche, #11277073910), 0.25 - 0.5 volume PCR template and 20 Units of the appropriate RNA polymerase (SP6 or T7; Promega, #P108 or #P207). Probe synthesis reactions were carried out at 37°C for two to four hours. For *beta tubulin*, a 702 bp long internal fragment was used for riboprobe synthesis. For *engrailed,* a 929 bp internal fragment partially covering the homeobox domain was used. The riboprobe against *COE* was generated from a 1626 bp long internal fragment covering the DNA binding domain and the TIG/IPT domain. All riboprobes were purified by precipitation using 0.1 volume of 3 M sodium acetate pH 5.2 and 3 volumes of absolute EtOH for 15 minutes, and subsequently centrifuged for 15 minutes at 16,000 RCF. All precipitation steps were carried out at room temperature. The resulting pellets were washed once in 75% EtOH, dried and dissolved in 10 μl water at 55°C. After quantification using a Nanodrop, 500 ng of riboprobe was denatured in 95% deionised formamide at 75°C for 10 minutes and qualitatively assessed by agarose gel electrophoresis. The remaining riboprobe solution was adjusted to a final concentration of 300 ng/μl using deionised formamide. In order to assess the affect of probe hydrolysis on WMISH signal, some riboprobes were also hydrolysed as described by [17].

### Probe hybridisation and antibody binding

All samples were incubated in hybridisation buffer for 15 minutes at room temperature before being heated to the hybridisation temperature of 55°C. The hybridisation buffer was then exchanged and incubated for an additional two hours. Each riboprobe in hybridisation buffer was denatured for ten minutes at 75°C and aliquoted into individual hybridisation reaction tubes for subsequent use in the robot. The hybridisation buffer on all samples was replaced by the riboprobe in hybridisation buffer and allowed to hybridise for 16 hours at 55°C using the following optimised concentrations of riboprobes: *beta tubulin* 100, 150 or 200 ng/ml; *engrailed* 500 ng/ml; *COE* 100 ng/ml or 300 ng/ml. Unbound probe was washed out with three washes in 4X wash buffer for 15 minutes each, three washes in 2X wash buffer for 15 minutes each, three washes in 1X wash buffer for 15 minutes each and one wash in 1X SSC + 0.1% Tween for 15 minutes, all performed at 55°C. Samples were then allowed to cool to room temperature and then washed twice in 1X SSC + 0.1% Tween for 15 minutes each. Two washes in maleic acid buffer (MAB) pH 7.5 were then performed for ten minutes each. All samples were then cooled to 10°C and incubated for three hours and 30 min in pre-cooled 2% block solution (Roche, #11096176001) in MAB with one exchange. Block solution was then replaced by block solution containing a 1/10,000 dilution of anti-DIG antibody conjugated to Alkaline Phosphatase (Roche, #11093274910) and incubated for five hours followed by a renewal of this solution and a further five hours incubation, all at 10°C. Samples were then allowed to warm to room temperature and unbound antibody was removed by 15 washes with PBTw for ten minutes each.

### Colour development and postprocessing

For colour development, samples were transferred into 1X Alkaline Phosphatase buffer with 0.1% (v/v) Tween 20 (APTw) and incubated with two ten minutes washes at room temperature. The 1X APTw buffer was replaced by the detection buffer and colour development was performed in the dark. Fluorescent signals were developed using the SIGMA FAST™ Fast Red TR/Napthol AS-MX Alkaline Phosphatase Substrate (Sigma, #F4648), prepared according to the manufactorer’s recommendations. All reactions were stopped by replacing the colour substrate solution with three five minutes washes in PBTw each, followed by two five minutes washes in 0.1 M Glycine pH 2. Samples for direct comparisons were stopped at the same time. After three further five minutes washes in PBTw, samples were postfixed in 4% (v/v) PFA in PBTw for at least two hours at room temperature or over night at 4°C. The fixative was removed by two five minutes washes with PBTw, followed by two washes with pre-warmed deionised water for each ten minutes at 37°C. Embryos were then dehydrated through a graded EtOH series (33%, 66%, twice with 100%) and stored at −20°C.

### Imaging

Prior to imaging samples were rehydrated through a graded EtOH series in PBTw (66%, 33%, twice with PBTw) and cleared at 4°C over night in 60% (v/v) glycerol. For “bulk” imaging (where a single image of tens of embryos gives an impression of the consistency of a given treatment) embryos were mounted in a 96 well plate with U-shaped bottom and imaged under a Zeiss stereo Discovery V8 microscope running Zeiss camera software Axio Vision Rel. 4.7. For images of individual embryos, samples were mounted on glass slides and photographed using a Zeiss Axio Imager Z1 microscope running Zeiss camera software Axio Vision Rel. 4.8. Images of all samples were captured using automatic settings for exposure and white balance. Images of individual embryos were also captured at different focal planes some of which were projected using Macnification version 2.0.1. All images were edited in Adobe Photoshop CS3 version 10.0.1 to achieve the optimal visual representation of each WMISH treatment and to facilitate qualitative comparisons. Each image was linearly adjusted for brightness, contrast and colour balance using the automatic function. These adjustments were applied to every pixel in each image and did not obscure, eliminate, or misrepresent any information.

Fluorescence detection was performed using a Zeiss LSM 510 Meta with the following microscope setup: HeNe 543 laser, HFT 488/543/633; NFT490; LP560. Both individual images and stacks were captured using the following settings: laser power of 2.9%; pinhole of 59 μm; amplifier gain of 1; amplifier offset and gain adjusted to the sample brightness; stack size of 1024 × 1024; scan speed and number of scans 7 and 4 respectively. For individual images the stack size was 2048 × 2048.

#### Solutions

**1X PBS (phosphate buffered saline):** 0.1 volume of 10X PBS stock (1.37 M NaCl; 27 mM KCl; 100 mM Na_2_HPO_4_.2H_2_O; 20 mM KH_2_PO_4_)

**1X PBTw (phosphate buffered saline + Tween 20):** 10% (v/v) of 10X PBS stock; 0.1% (v/v) Tween-20

**2.5% NAC (N-acetyl cysteine):** 50% (v/v) of 5% (w/v) NAC in 1X PBS

**4% PFA (paraformaldehyde):** 25% (v/v) of 16% (w/v) PFA pH 7–8; 1X PBS or 1X PBTw

**0.5% SDS (sodium dodecyl sulfate)**: 2.5% (v/v) of 20% (w/v) SDS; 1X PBS

**0.1X reduction solution:** 0.1% (v/v) Tergitol (NP40); 0.05% (v/v) sodium dodecyl sulfate (SDS); 5 mM dithiothreitol (DTT)

**1X reduction solution:** 1% (v/v) Tergitol (NP40); 0.5% (v/v) SDS, 50 mM DTT

**33% EtOH (ethanol):** 33% (v/v) volume of absolute EtOH in PBTw

**66% EtOH (ethanol):** 66% (v/v) volume of absolute EtOH in PBTw

**Pro-K (Proteinase K):** Diluted from 10 mg/ml stock using PBTw

**2 mg/ml glycine pH 2**: Diluted from 100 mg/ml stock using PBTw

**TEA (triethanolamine):** 1% (v/v) TEA diluted in 1X PBTw

**TEAAA (triethanolamine + acetic anhydride):** 1% (v/v) TEA; 0.3% (v/v) acetic anhydride diluted in 1× PBTw

**Hybridisation buffer**: 25% (v/v) 20X SSC stock (3 M NaCl; 0.3 M trisodium citrate dihydrate); 5 mM ethylene diamine tetra-acetic acid (EDTA) from 500 mM stock pH 8.0; 0.5 volume deionised formamide; 100 μg/ml Heparin from 100 mg/ml stock; 0.1% (v/v) Tween-20; 1X Denhardt’s from 100X stock (2% (m/v) Ficoll type 400; 2% (w/v) polyvinylpyrrolidone K30; 2% (w/v) bovine serum albumin); 100 μg/ml single-stranded salmon sperm DNA from 10 mg/ml stock

**4X wash:** 20% (v/v) 20X SSC stock; 50% (v/v) formamide; 0.1% (v/v) Tween-20

**2X wash**: 10% (v/v) 20X SSC stock; 50% (v/v) formamide; 0.1% (v/v) Tween-20

**1X wash:** 5% (v/v) 20X SSC stock; 50% (v/v) formamide; 0.1% (v/v) Tween-20

**MAB (maleic acid buffer):** 0.1 M maleic acid from 1 M stock pH 7.5; 0.15 M NaCl from 5 M stock

**Block solution:** 2% (v/v) block from 10% (w/v) stock in MAB

**Antibody solution:** AP-conjugated anti-DIG fab fragments diluted 1/10,000 in block solution

**1X APTw (Alkaline Phosphatase buffer + Tween 20):** 20% (v/v) 5X AP buffer stock (0.5 M Tris pH 9.5 from 1 M stock; 0.5 M NaCl from 5 M stock); 0.1% (v/v) Tween-20

**Colour detection buffer:** 1X APTw; 50 mM MgCl_2_ from 1 M stock; 450 μg/ml NBT from 100 mg/ml stock in DMF; 175 μg/ml BCIP from 50 mg/ml stock in water

**Colour detection buffer + PVA (polyvinyl alcohol):** 1X APTw; 50 mM MgCl_2_ from 1 M stock; 450 μg/ml NBT from 100 mg/ml stock in DMF; 175 μg/ml BCIP from 50 mg/ml stock in water; all diluted in 10% (w/v) PVA in water

**Fluorescent colour detection buffer:** 0.1 M Tris; 1 mg/ml Fast Red TR; 0.4 mg/ml Napthol AS-MX; 0.15 mg/ml Levamisol; final pH 7.9-8.5

**Stop solution:** 0.1 M glycine pH 2.2 from 1 M stock; 0.1% (v/v) Tween-20.

**PFA:** 4% (v/v) PFA in 1x PBTw

**60% glycerol:** 60% glycerol (v/v) in water

## Results and discussion

Previously described WMISH protocols for molluscan embryos and larvae did not yield consistent or satisfactory WMISH signals in *L. stagnalis* [14,21,22]. Therefore, we focused on a few key steps of sample preparation we believed to cause background, weak WMISH signals and non-specific staining. Note that in this work we did not explore the effect of hybridisation temperature on the final result. Initial experiments with the probes used in the present study revealed that 55°C produced consistent and acceptable results, allowing us to focus on systematically optimising other parameters. Of course, hybridisation temperature should be empirically optimised for every probe and has the potential to significantly improve or impair the final result. A summary of the treatments we found to generate the clearest WMISH signals for each developmental stage (a “protocol at a glance”) is provided in Additional file 2. The results of control experiments using no antibody and no riboprobe (which generated no signals) are provided in Additional file 3.

### The effect of NAC treatment

**Figure 2:**
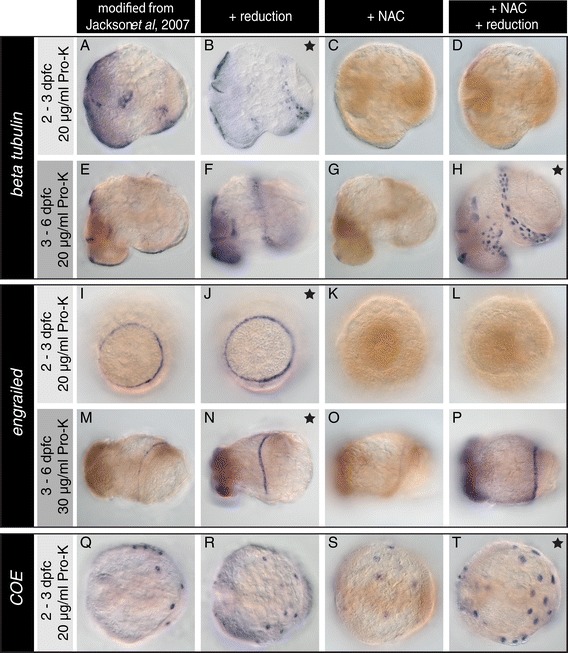
**Overview of WMISH signals generated after pre-hybridisation treatment with NAC and/or reduction.** *L. stagnalis* larvae of different ages were subjected to a WMISH protocol similar to that described by Jackson *et al.* [22] **(A, E, I, M and Q)**. This protocol was then modified by the addition of a reduction treatment **(B, F, J, N and R)**, a NAC treatment **(C, G, K, O and S)** or a combination of both NAC and reduction treatment **(D, H, L, P and T)**. Using this set of pre-hybridisation treatments, the optimal sample preparation regime for WMISH varies with respect to the target gene and the developmental stage. For *engrailed* and *beta tubulin* in younger larvae, the samples that underwent a reduction treatment display the best signal to noise ratio **(B, J and N)**. Excess background that is revealed by reduced samples for *COE* and *beta tubulin* in older larvae **(F and R)** is diminished by a treatment with NAC **(H and T)**. Black stars indicate optimal results after sample preparation involving NAC treatment and reduction. Panels **A** to **H** show larvae from a lateral perspective with the shell field oriented to the right. Larvae in **I** to **P** are viewed from dorsal and **Q** to **T** are viewed from apical.

The fluid that bathes *L. stagnalis* larvae during their encapsulated development is characterised by a high viscosity and adheres to the embryo following de-capsulation. An incubation step with the mucolytic reagent NAC apparently leads to a superior preservation of the overall morphology (Figure 2 and Additional file 3C *cf.* E and 3I *cf.* K). However, treatment with NAC resulted in a significant reduction of signal intensity for all ages and genes that we investigated (Figure 2C, G, K, O and S). However, when NAC treatment was combined with a reduction step this effect was reversed for some combinations of riboprobe and developmental stage (Figure 2H, P and T). The combined NAC and reduction treatment gave the best signal to noise ratio for *beta tubulin* in three to six dpfc larvae (Figure 2H), and for *COE* in two to three dpfc old larvae (Figure 2T). This is in contrast to the situation for *engrailed* in all investigated ages: under the appropriate reduction treatment, omitting the NAC generated a better signal to noise ratio (Figure 2J and N) than including it (Figure 2K, L, O and P).

Our overall impression of treating *L. stagnalis* larvae with NAC is that this treatment may be beneficial in combination with a reduction step when working with probes, which tend to generate non-specific background. The signal diminishing effect of NAC is in contrast to the situation in the planarian *Schmidtea mediterranea*. Here, a NAC treatment is used to remove the planarian’s surrounding mucous layer and generally increases the WMISH staining intensity, at least when combined with a permeabilisation step using SDS or DTT [17]. In *L. stagnalis*, NAC may be removing the intra-capsular fluid, however it appears that in our hands NAC is most likely reducing WMISH signal strength by significantly inhibiting the activity of Pro-K; larvae that were incubated in Pro-K and 1% NAC at the same time did not show any signs of compromised morphology, while larvae in control reactions with Pro-K but with less (0.1%) or without NAC were digested (Additional file 4).

### Treatment with DTT and detergents (reduction)

A treatment using a solution containing DTT and the detergents SDS and NP-40 following fixation greatly increased WMISH signal intensity for all investigated genes and developmental stages (Figure 2). The best WMISH signal for *beta tubulin* in four to six dpfc old larvae was achieved using a combination of NAC and reduction (Figure 2H). This suggests that the reduction treatment might represent a highly effective permeabilisation approach. However, this was at the cost of all material becoming highly fragile until dehydrated in ethanol. Reduced samples were also more likely to reveal unspecific background staining (Figure 2F, N and R).

### SDS treatment

**Figure 3:**
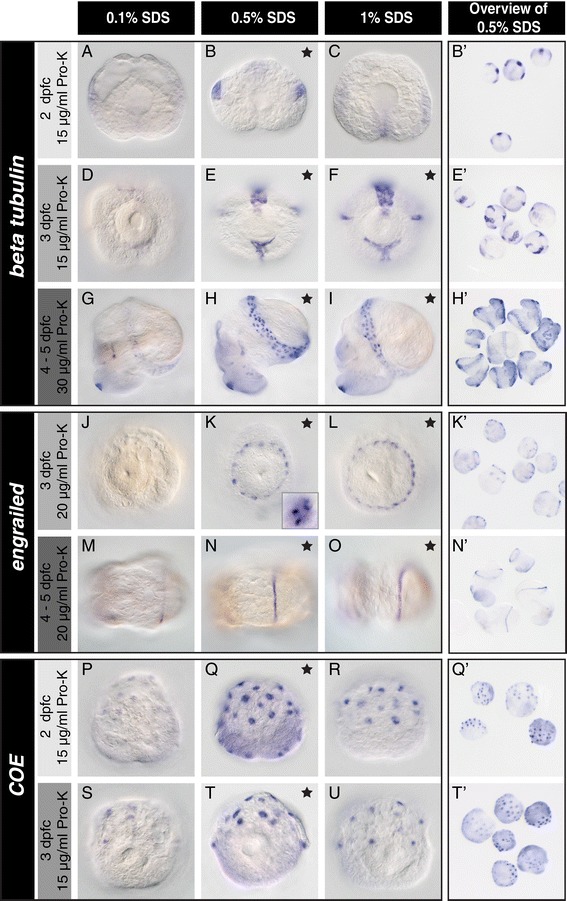
**A pre-hybridisation treatment with different SDS concentrations significantly affects the WMISH signal.** *L. stagnalis* larvae of different ages were subjected to pre-hybridisation treatments with varying amounts of SDS and then hybridised with anti-sense probes to *beta tubulin* **(A-I)**, *engrailed* **(J-O)** and *COE* **(P-U)**. For all genes and larval ages, treatment with 0.1% SDS did not generate consistent or strong WMISH signals **(A, D, G, J, M, P and S)**. Treatments with both 0.5% and 1% SDS produced strong WMISH signals for *beta tubulin* and *engrailed* in larvae aged three to five days post first cleavage (dpfc), with high spatial resolution (inlet in **K**). For *COE* 0.5% SDS outperformed the 1% SDS treatment (**T** vs. **U**). Black stars indicate optimal treatments. Note that some treatments produced equally good results. The most consistent results (defined as constantly good signals among genes and ontogenetic stages with little variation between individuals within an experiment) were achieved with 0.5% SDS (examples shown in **B’**, **E’**, **H’**, **K**’, **N**’, **Q**’ and **T’**). Larvae in **A-C** and **M-R** are shown from an apical perspective, larvae in **D-F** are viewed ventrally, **G-I** laterally and **J-L** and **S-U** dorsally.

Between one and five dpfc old embryos and larvae of *L. stagnalis* were treated with different amounts of the anionic detergent SDS prior to hybridisation (Figure 3). A permeabilising treatment with 0.1% SDS did not produce strong WMISH signals for all studied genes and larval ages (Figure 3A, D, G, J, M, P and S) whereas treatments with higher concentrations of SDS generated strong WMISH signals. For two of the genes we studied here, *beta tubulin* and *engrailed*, treating larvae between three to five dpfc with 0.5% or 1% SDS produced equally good results. In contrast, the staining was more intense after treatment with 0.5% SDS than with 1% SDS for *COE* (Figure 3Q vs. R and T vs. U) as well as for *beta tubulin* in two dpfc old larvae (Figure 3B vs. C), which may suggest a loss of the target transcripts due to excess permeabilisation of these younger stages. Additionally, embryos between one and two dpfc tend to adhere to plastic surfaces in 1% SDS. While in other animal systems SDS is commonly used at a concentration of 1% [18-20], in *L. stagnalis* strong WMISH signals were most consistently achieved with 0.5% SDS across different genes and ontogenetic stages.

The WMISH signals for *engrailed*, *COE* and *beta tubulin* (the latter at least in younger larvae) in SDS-treated larvae reveal equally good or superior signal intensities compared to reduced or NAC-treated and reduced larvae (Figure 2*cf.* Figure 3). The *engrailed* signal in the shell field of SDS-treated larvae also clearly exhibits a significantly improved spatial resolution compared to the signal in reduced larvae (Figure 2J *cf.* Figure 3K). In terms of ease of handling, non-specific background and consistency of WMISH signal among the different genes, the SDS treatment is our recommended sample preparation strategy. A reduction treatment might increase the signal intensity in WMISH experiments against genes expressed in older larvae (Figure 2H), but should then be performed in parallel to the SDS treatment to control for a possible loss of spatial resolution.

### Enzymatic permeabilisation with Pro-K

The optimal Pro-K concentration for tissue permeabilisation and mRNA target unmasking depends on the incubation temperature, incubation time and the ontogenetic stage of the material. In general, under-treatment yields weak WMISH signals while over-treatment increases background staining and leads to a compromised tissue morphology. We tested a wide range of Pro-K concentrations in combination with different pre-hybridisation treatments and found that treatment with Pro-K at concentrations of 0–2 μg/ml drastically reduced signal intensity while treatment at concentrations of 40–50 μg/ml compromised tissue integrity (data not shown). Therefore, we finally used concentrations of 1–15 μg/ml for one to two dpfc old larvae, 5–20 μg/ml for two to three dpfc old larvae and 5–40 μg/ml for larvae between three and five dpfc in combination with different SDS concentrations (data not shown). For embryos between one and two dpfc we found the highest concentration of Pro-K of 15 μg/ml gave the strongest signals (Figure 3B and Q). For two and three dpfc larvae, 15 μg/ml or 20 μg/ml Pro-K were suitable (Figure 3E, F, K, L and T). In larvae between three and five dpfc the best signal to noise ratio was achieved with 30 μg/ml for *beta tubulin* (Figure 3H and I) and 20 μg/ml for *engrailed* (Figure 3N and O). *COE* is apparently not expressed in larvae between three and five dpfc.

### Removal of non-specific background with triethanolamine + acetic anhydride (TEAAA)

**Figure 4:**
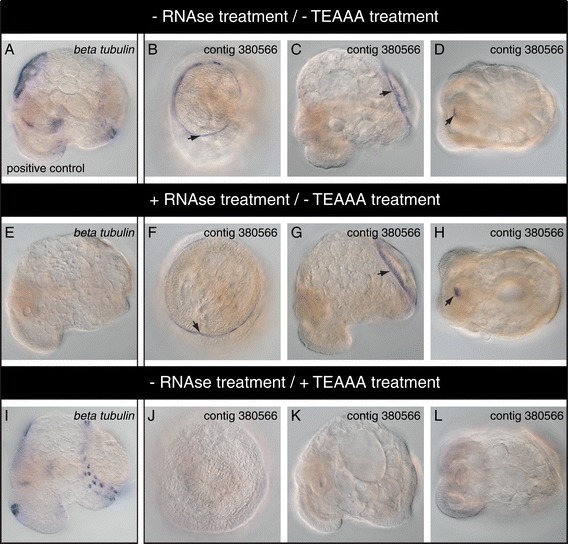
**Non-specific probe binding to the shell field and radular is eliminated by treatment with TEAAA.** We observe a well-defined and consistent WMISH stain for a variety of probes (represented here with a probe against the gene “contig 380566”) in the periphery of the shell field (arrows in **B** and **C**) and in the radular sac (arrow in **D**). Probes against other genes (for example *beta tubulin*) do not produce these patterns **(A)**. The stain in the shell field periphery and the radular remains following a pre-hybridisation treatment with RNAse **(F-H)**, while the specific signal against *beta tubulin* is abolished **(E)**. This indicates that the signals in the shell field and the radular are the result of non-specific probe binding. Treatment with TEAAA abolishes this non-specific stain **(J-L)**, while the specific signal against *beta tubulin* remains unaltered **(I). B**, **F** and **J** are dorsal views. **A**, **C**, **E**, **G**, **I** and **K** are lateral views of larvae with the shell gland oriented to the right. **D**, **H** and **L** are ventral views. Panels **C**, **G** and **K** are reflected about the vertical axis for consistency of presentation.

In preliminary experiments using a wide variety of riboprobes to different genes we obtained a strong, well-defined WMISH signal located at the periphery of the shell field and in the radular sac (Figure 4, black arrows). To determine whether these staining patterns represented genuine probe/target hybridisation events, or non-specific binding of the probe, we treated fixed larval material with RNAse A prior to hybridisation. As a control, all expected *beta tubulin* staining was abolished following this RNAse treatment, confirming the degradation of all target mRNA (Figure 4E). In RNAse treated embryos hybridised with probes that generated the spurious shell field and radula patterns this signal was still present (Figure 4F-H), indicating these WMISH patterns represent high affinity binding of the riboprobe to molecules other than RNA. In order to address this background staining, we assessed the effect of a triethanolamine + acetic anhydride (TEAAA) treatment. Treatment of biological substrates with TEAAA is a common practice for many WMISH protocols, and decreases non-specific binding of labelled probes through the acetylation of polar and charged groups [23]. For *L. stagnalis*, treatment with TEAAA following Pro-K digestion successfully abolished the non-specific WMISH signal in the larval shell and in the radular sac (Figure 4J-L). Due to its strength, consistency and spatial definition, this tissue-specific background stain is particularly likely to be misinterpreted as genuine WMISH signal. A good example of this is *engrailed*, which is genuinely expressed in the shell field periphery (Figure 3J-O) and also produces the shell field background stain (data not shown). Our riboprobe against *engrailed* (synthesised multiple times) covers the same region as the probe used in a recent study that possibly produced the same background staining [14]. Therefore, treatment with TEAAA appears to be critical for the correct interpretation of genes with expression patterns associated with the shell gland and shell field.

General background staining and its elimination by TEAAA can also be observed for samples that underwent a NAC (Figure 2) or SDS treatment (Figure 3). For some genes, the TEAAA treatment can be shortened by one incubation step in TEAAA instead of two incubation steps (Additional file 5).

### The effect of antibody concentration, PVA and riboprobe hydrolysis

**Figure 5:**
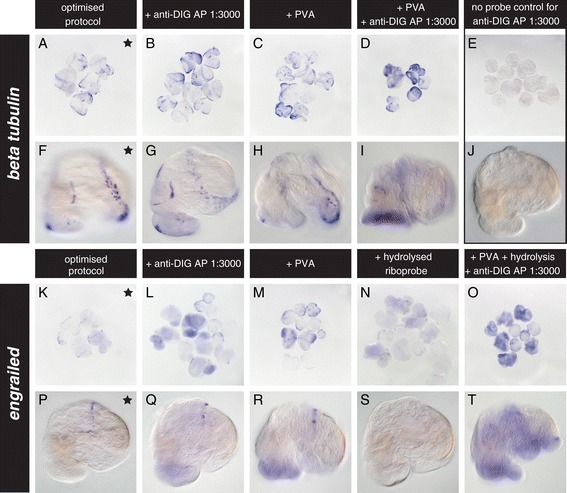
**Our optimised WMISH protocol is not improved by more antibody, PVA or hydrolysed riboprobes.** Larvae three to four days post first cleavage (dpfc) were subjected to our optimised WMISH protocol **(A, F, K and P)**. Using a *beta tubulin* probe, we investigated the effect of increasing the amount of anti-DIG antibody **(B, G, L and Q)**, the addition of PVA to the colour detection solution **(C, H, M and R)** and the combination of more antibody and PVA **(D and I)**. We also assessed the effect of hydrolysing the *engrailed* riboprobe individually **(N and S)** and in combination with a higher antibody concentration and the use of PVA **(O and T)**. None of these modifications generated superior WMISH results to our baseline protocol. Samples incubated in more antibody and developed with PVA showed slightly more intense signals, but a lower signal to noise ratio **(B, C, G, H, L, M, Q and R)**. PVA also appeared to compromise the morphological integrity of older larvae **(H, I and R)**. Signals generated by the hydrolysed *engrailed* probe were much fainter and were partially masked by an increase in general background staining **(N, O, S and T)**. The optimal treatment **(A, F, K, and P)** is indicated by a black star. Control WMISH experiments lacking a riboprobe and using the increased antibody concentration do not reveal any staining **(E and J)**. All images of individual larvae are lateral views.

Since the dilution of AP-conjugated anti-DIG antibody in our optimised protocol (1/10,000) is lower than described in many WMISH protocols, we assessed the effect of increasing the concentration of antibody to 1/3,000. While the signal intensities of *beta tubulin* and *engrailed* expression were slightly higher, more overall non-specific background staining was also evident (Figure 5B, G, L and Q).

A common approach to improve WMISH sensitivity and reduce background staining is through the addition of PVA to the colour detection solution. This is thought to increase the local concentration of the colour reaction product by limiting its diffusion [24]. A direct comparison of WMISH colour development in *L. stagnalis* with and without PVA did not reveal a significant increase in the signal intensities, but rather a lower signal to noise ratio for both *beta tubulin* and *engrailed* (Figure 5C, H, M and R). Furthermore, the morphological integrity of especially older larvae was compromised (Figure 5H).

An alternative strategy to increase WMISH signal sensitivity is to hydrolyse the riboprobe before use [23,24]. Hydrolysing the riboprobe into smaller fragments is thought to facilitate better tissue penetration and to improve hybridisation kinetics (reviewed in [23,25]). We specifically tested whether a hydrolysed riboprobe generates an improved *engrailed* signal and found that instead both the signal specificity (as revealed by the lack of the shell field lining expression pattern, Figure 5S) and overall intensity were reduced compared to non-hydrolysed control reactions (Figure 5P vs. S). Reduced signal sensitivity derived from hydrolysed riboprobes has been previously reported [26], highlighting the necessity to test the optimal probe preparation strategy for each gene individually [27].

To summarise these treatments, neither more antibody nor the use of PVA or hydrolysed riboprobes generated WMISH signals with higher sensitivity and/or signal to noise ratios.

### Fluorescent WMISH signal detection

**Figure 6:**
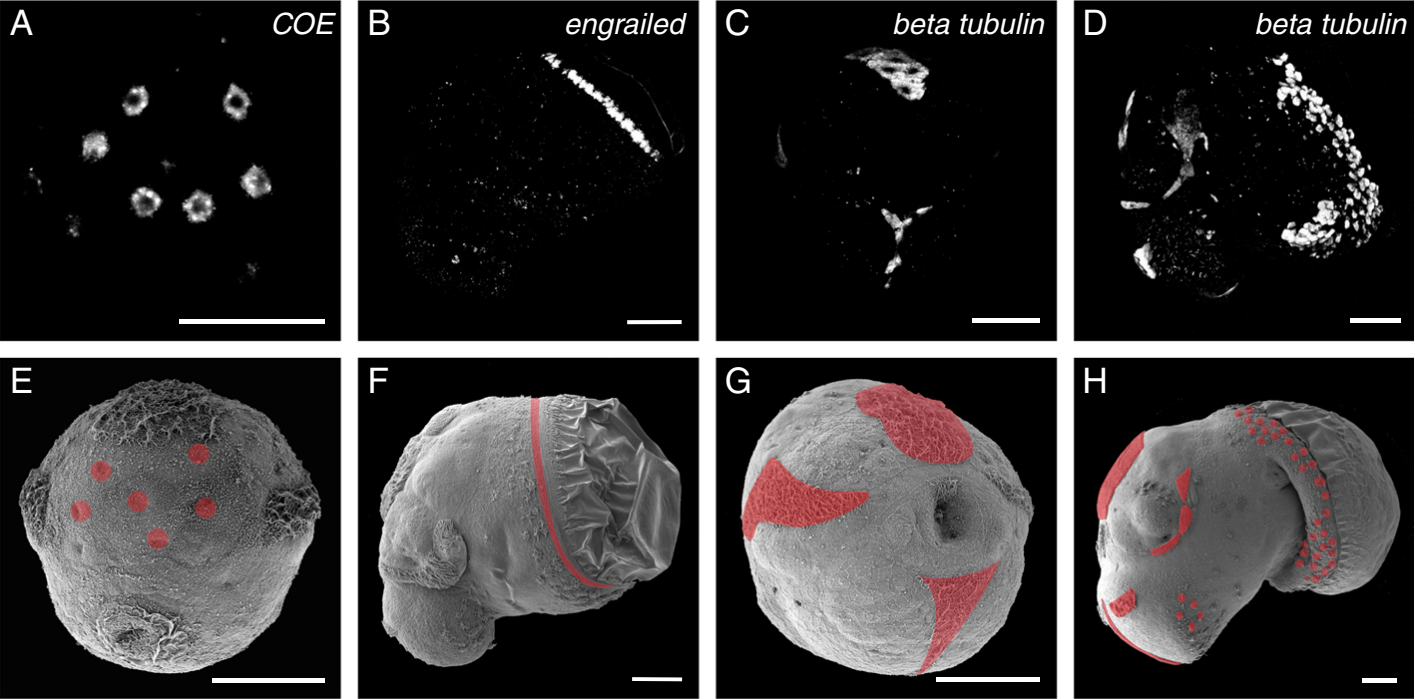
**Our WMISH protocol is suitable for fluorescent signal detection.** The expression of *COE* **(A)**, *engrailed* **(B)** and *beta tubulin* **(C and D)** in larvae treated with 0.5% SDS was detected using the fluorescent substrate Fast Red **(A-D)**. Panel **E** is a scanning electron micrograph of a 62 hours post first cleavage (hpfc) old larva with the approximate localisation of the *COE* expression indicated in red. Panel **F** is a lateral perspective of a 90 hpfc old larva with indicated *engrailed* expression. Panels **G** and **H** show the positions of *beta tubulin* expression in a 57 hpfc old larva (**G**, ventral perspective) and a 100 hpfc old larva (**H**, lateral perspective). Panels **B-D** are projections of confocal laser scanning micrographs. Panels **B**, **D** and **H** are reflected about the vertical axis for consistency of presentation. All scale bars are 50 μm.

The optimised protocol that we have identified also allows for the visualisation of fluorescent signals using a confocal microscope. This method of detection provides a much higher spatial resolution than colorimetric methods. Using probes against all three of the genes employed in this study we were able to develop fluorescent signals using Fast Red (Figure 6).

### The effect of storing fixed material in Ethanol vs. Methanol

Methanol (MeOH) is used to dehydrate and store fixed embryonic and larval material at low temperatures (−20°C) in many WMISH protocols. Due to the high toxicity of MeOH relative to EtOH we assessed the effect of storing fixed *L. stagnalis* larvae in MeOH vs. EtOH on the WMISH signal generated by *beta tubulin*, *engrailed* and *COE*. We found no consistent or significant difference with respect to any of the signals generated (data not shown).

## Conclusions

Our study provides an optimised whole mount *in situ* hybridisation protocol for early larval stages of the mollusc *L. stagnalis*. Using a variety of pre-hybridisation treatments we have identified a set of conditions that allow for high WMISH signal intensity and consistency in colorimetric as well as fluorescent WMISH. These include a treatment with 0.5% SDS, treatment of one to two dpfc larvae with 15 μg/ml Pro-K, two to three dpfc larvae with 15–20 μg/ml Pro-K and three to five dpfc larvae with 20–30 μg/ml, followed by treatment with TEAAA. We also demonstrate that non-specific shell field and radula staining can easily be abolished with this TEAAA treatment. In our experience, every riboprobe/developmental stage combination benefits from an individualised protocol, which needs to be empirically determined. Nonetheless we believe that this WMISH protocol should serve as a baseline method from which consistent and clearly visible patterns of gene expression can be obtained. This method should serve to raise the profile of *L. stagnalis* as a tractable experimental molluscan model, a niche that is currently underpopulated.

## Additional files

Additional file 1:
**Primer sequences used to isolate gene fragments for riboprobe syntheses.**
Additional file 2:
**WMISH “protocol at a glance”.**
Additional file 3:
**Control experiments for the optimised sample preparations.** Control WMISH experiments lacking riboprobe (A-F) or antibody (G-N) demonstrate the absence of any non-specific colour reaction for samples treated with SDS (A, B, G, H, M and N) or with reduction solution (C, D, I and J) as well as reduced + NAC treated samples (E, F, K and L) for about three dpfc old larvae (A, C, E, G, I, K and M) and about five dpfc old larvae (B, D, F, H, J, L and N). Panels A to L were colour-developed using NBT/BCIP as colour substrate and panels M and N were developed using Fast Red. All embryos are shown from a lateral perspective. Scale bars are 50 μm (A, C, E, G, I, K and M) or 100 μm (B, D, F H, J, L and N). Panels C, E, G and H are reflected about the vertical axis for consistency of presentation. Additional file 4:
**Proteinase-K activity is inhibited by NAC. Larvae incubated in Pro-K without NAC (A-D) or in 0.1% NAC (E-H) are almost completely digested after 4 and 22 hours of incubation respectively.** In contrast, larvae incubated in Pro-K with 1% NAC (I-L) do not show any signs of Pro-K digestion and maintain their morphology even over extended incubation times (L). All larvae are about 4 days post first cleavage old. All images are to the same scale shown in L (1 mm). Additional file 5:
**A shortened treatment with TEAAA is sufficient to minimise non-specific probe binding in SDS-treated samples.** The background stain in the shell field periphery (identified in Figure 3) is also observed for SDS-treated samples (arrows in A), as represented by a probe against the gene “contig 380566”. Note that after treatment with SDS, the protonephridia are stained (arrow in B). Both non-specific WMISH stains are strongly reduced after one incubation step in TEAAA (D-F) and disappear after two incubation steps (G-I). Larvae in A, D and G are shown from a dorsal perspective, and larvae in B, E and H are viewed from lateral. Panels B, E and H are reflected about the vertical axis for clarity of presentation.
