# Supplementary material for: An optimised whole mount in situ hybridisation protocol for the mollusc Lymnaea stagnalis
Source: BMC Dev Biol. 2015 Mar 28;15:19. doi: 10.1186/s12861-015-0068-7 (PMC4379745; doi:10.1186/s12861-015-0068-7)
Supplement: Additional file 2: — WMISH “protocol at a glance”. [file 12861_2015_68_MOESM2_ESM.docx]

**Additional file 2. WMISH “protocol at a glance”**

We perform all sample preparation steps (Day 1) in 12 well tissue culture tissues using 2 ml of each solution. Gentle manual agitation encourages the larvae to migrate to the centre of the well making aspiration of the liquid from the edge of the well easier. This also allows for the removal of any physically damaged material under a stereomicroscope. All steps are carried out at room temperature unless otherwise indicated.

**Day 1: Fixation, SDS treatment and dehydration**

1. Free capsules from jelly string, and then larvae from their capsules.
2. Transfer larvae into 4% PFA in PBS and incubate for 30 min with occasional gentle agitation.
3. Remove fixative and wash larvae for 5 min in PBTw.
4. Remove PBTw and incubate larvae for 10 min in 0.5% SDS in PBS without agitation.
5. Rinse with one exchange of PBTw without agitation. Either continue to the next step or proceed immediately to Day 2.
6. Exchange for 33% EtOH in PBTw and let sit for 5-10 min.
7. Exchange for 66% EtOH in PBTw and let sit for 5-10 min.
8. Exchange for 100% EtOH and let sit for 5-10 min.
9. Replace with fresh 100% EtOH and store at -20°C until needed.

**Day 2: Rehydration, Pro-K, TEA, pre-hybridisation and hybridisation**

1. Replace 100% EtOH with 66% EtOH in PBTw and let sit for 5-10 min.
2. Exchange for 33% EtOH in PBTw and let sit for 5-10 min.
3. Exchange for PBTw and incubate for 5-10 min. Repeat.
4. Incubate larvae in Pro-K using the following regimes without agitation:

- 1-2 dpfc in 15 µg/ml Pro-K in PBTw for 10 min at RT.
- 2-3 dpfc with 20 µg/ml Pro-K for 10 min at RT.
- 3-5 dpfc with 30 µg/ml Pro-K for 10 min at RT.

1. Replace Pro-K solution with 2 mg/ml Glycine and incubate for 5 min without agitation.
2. Exchange for fresh glycine and incubate for 5 min without agitation.
3. Rinse with PBTw.
4. Replace with 1% TEA and let sit for 5 min without agitation.
5. Replace with fresh 1% TEA and incubate for 5 min without agitation.
6. Exchange for TEAAA and incubate for 5 min without agitation. *Note: prepare TEAAA immediately before use. Optional: repeat this step to eliminate persistent specific background staining.*
7. Postfix larvae for 15-20 min in 4% PFA in PBTw.
8. Wash three times with PBTw for 5-10 min.
9. Warm hybridisation buffer to room temperature.
10. Replace PBTw with pre-warmed hybridisation buffer and incubate for 15 min at room temperature.
11. Heat all samples to hybridisation temperature.
12. Exchange hybridisation buffer once and incubate for two hours at hybridisation temperature.
13. Dilute riboprobes in hybridisation buffer at room temperature.
14. Denature riboprobe dilutions for 10 min at 75°C.
15. Replace hybridisation buffer with riboprobe dilutions.
16. Incubate overnight (16+ hours) at hybridisation temperature.

**Day 3: Hot washes, antibody block and antibody incubation**

1. Pre-warm all wash solutions to hybridisation temperature.
2. Wash all samples three times in 4X wash buffer for 15 min each at hybridisation temperature.
3. Wash all samples three times in 2X wash buffer for 15 min each at hybridisation temperature.
4. Wash all samples three times in 1X wash buffer for 15 min each at hybridisation temperature.
5. Wash all samples once with 1X SSC + 0.1% Tween at hybridisation temperature.
6. Allow samples to cool to room temperature.
7. Wash twice in 1X SSC + 0.1% Tween for each 15 min.
8. Replace with MAB and let sit for 10 min. Repeat.
9. Cool samples to 10°C.
10. Pre-cool block solution.
11. Replace with cold block solution and incubate for 1.5 h.
12. Exchange for fresh cold block solution and incubate for 1.5 h.
13. Replace with cold antibody solution and incubate for several hours.
14. Exchange for fresh cold antibody solution and incubate overnight.

**Day 4: Antibody washes and colour development**

1. Allow samples to warm to room temperature.
2. Wash 15 times with PBTw for 10 min each.
3. Replace with 1X APTw and incubate for 10 min.
4. Exchange for fresh 1X APTw and incubate for 10 min.
5. Replace with colour detection buffer and monitor until the signal to background ratio is optimal.
6. Remove colour substrate by three washes with PBTw for 5 min each.
7. Stop the colour reaction by two washes with 0.1 M Glycine pH 2 for 5 min each.
8. Remove stop solution by three washes with PBTw for 5 min each.
9. Postfix samples in 4% PFA in PBTw for at least 2 h at room temperature or at 4°C over night.
10. Wash twice with PBTw for each 10 min.
11. Wash twice in pre-warmed to 37°C deionised water and incubate for 10 min at 37°C. *Note: this step removes residual salts that might precipitate during the following dehydration.*
12. Samples can now be cleared in 60% glycerol and either mounted for photo documenting or stored at 4°C. Alternatively, samples can be dehydrated and stored in 100% EtOH at -20°C. *Note: a dehydration in EtOH removes slight overall background staining.*
