# Supplementary figures and images for: An optimised whole mount in situ hybridisation protocol for the mollusc Lymnaea stagnalis
Source: BMC Dev Biol. 2015 Mar 28;15:19. doi: 10.1186/s12861-015-0068-7 (PMC4379745; doi:10.1186/s12861-015-0068-7)

+ SDS

+ reduction

+ NAC / reduction

no probe control

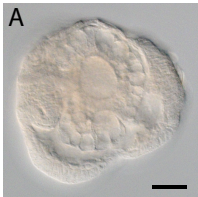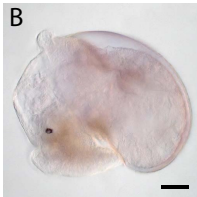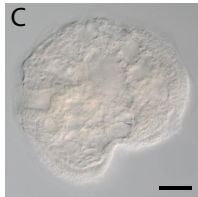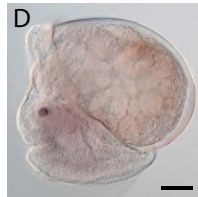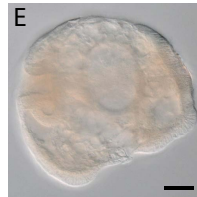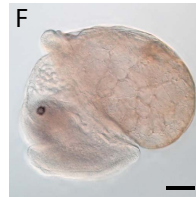

no antibody control

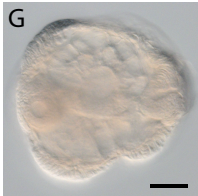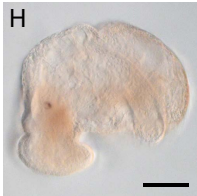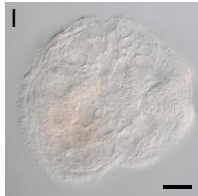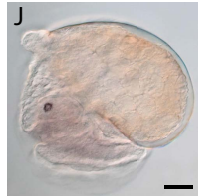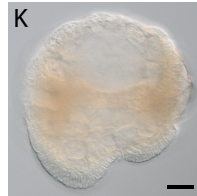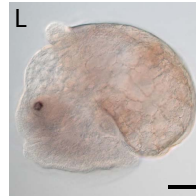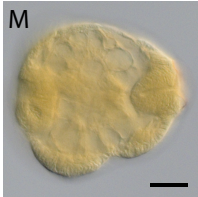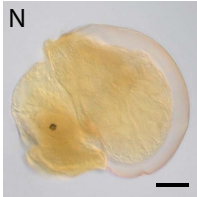

Supplement: Additional file 3: — Control experiments for the optimised sample preparations. Control WMISH experiments lacking riboprobe (A-F) or antibody (G-N) demonstrate the absence of any non-specific colour reaction for samples treated with SDS (A, B, G, H, M and N) or with reduction solution (C, D, I and J) as well as reduced + NAC treated samples (E, F, K and L) for about three dpfc old larvae (A, C, E, G, I, K and M) and about five dpfc old larvae (B, D, F, H, J, L and N). Panels A to L were colour-developed using NBT/BCIP as colour substrate and panels M and N were developed using Fast Red. All embryos are shown from a lateral perspective. Scale bars are 50 μm (A, C, E, G, I, K and M) or 100 μm (B, D, F H, J, L and N). Panels C, E, G and H are reflected about the vertical axis for consistency of presentation. [file 12861_2015_68_MOESM3_ESM.pdf]

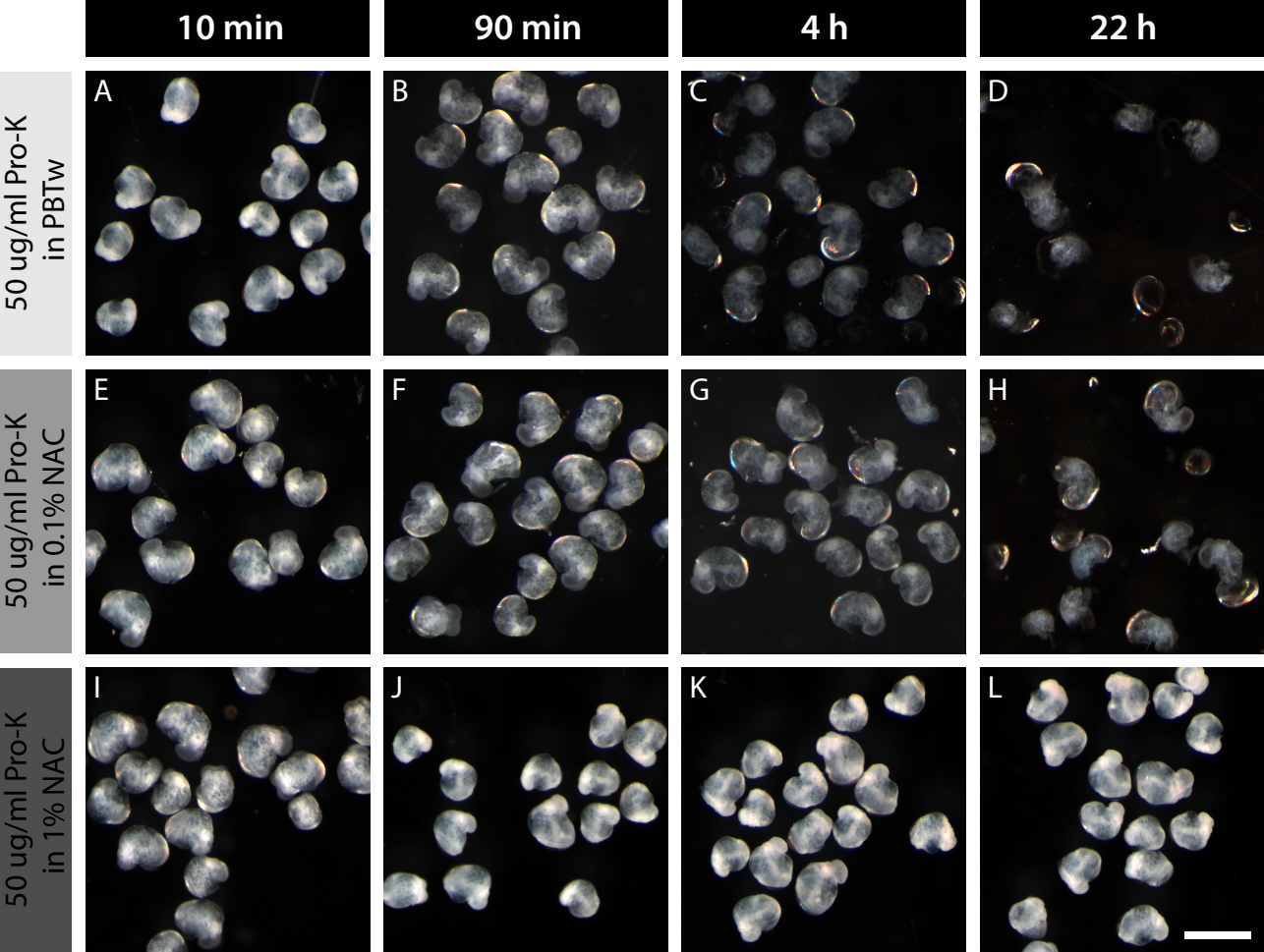

Supplement: Additional file 4: — Proteinase-K activity is inhibited by NAC. Larvae incubated in Pro-K without NAC (A-D) or in 0.1% NAC (E-H) are almost completely digested after 4 and 22 hours of incubation respectively. In contrast, larvae incubated in Pro-K with 1% NAC (I-L) do not show any signs of Pro-K digestion and maintain their morphology even over extended incubation times (L). All larvae are about 4 days post first cleavage old. All images are to the same scale shown in L (1 mm). [file 12861_2015_68_MOESM4_ESM.pdf]

## No TEAAA treatment

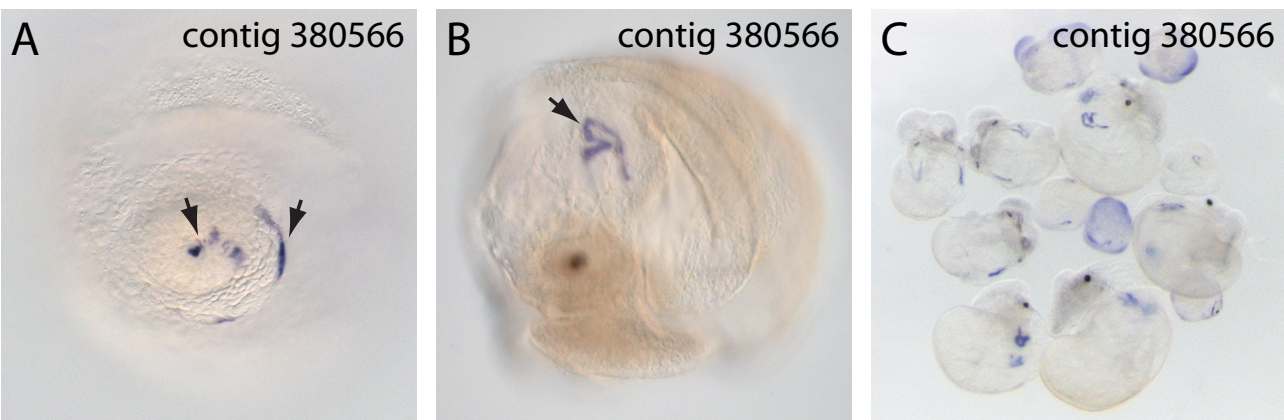

## One TEAAA treatment

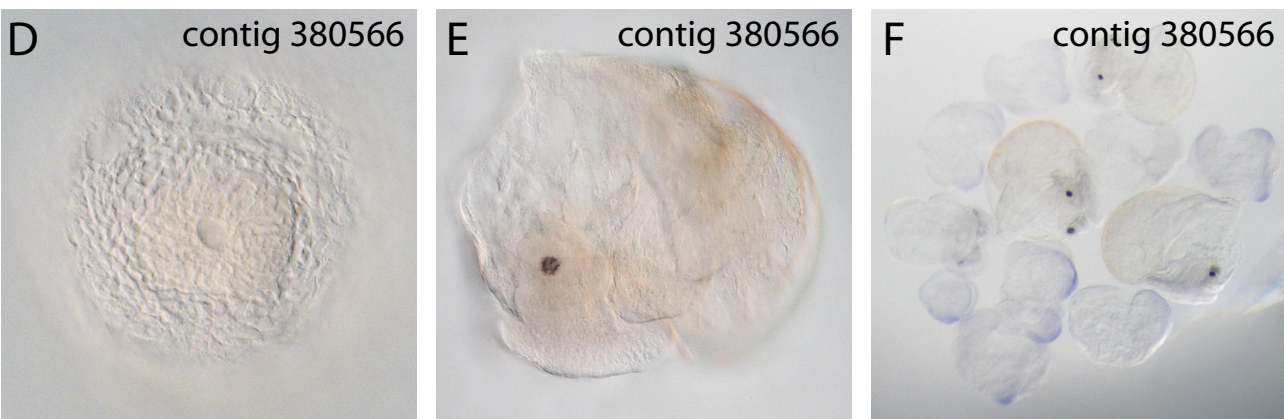

## Two TEAAA treatments

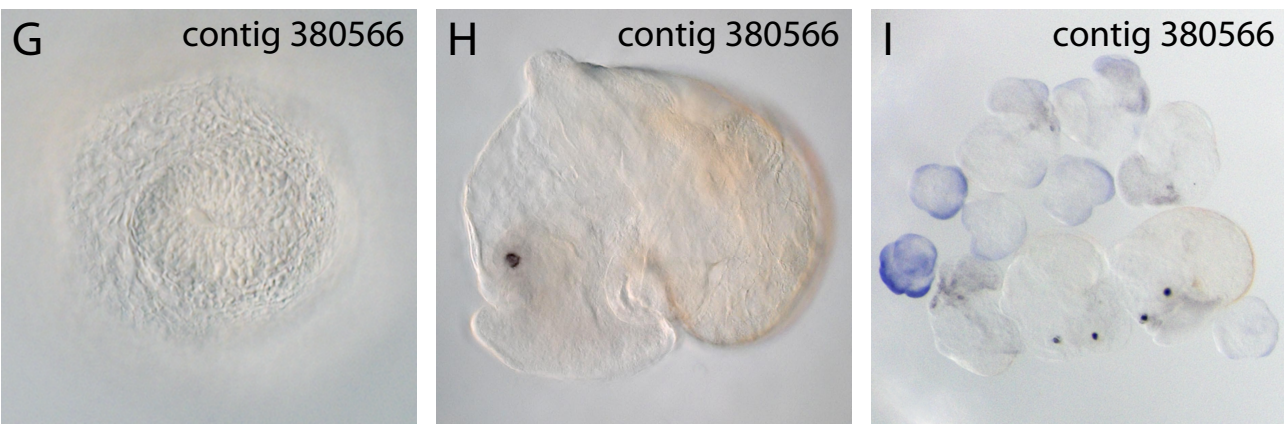

Supplement: Additional file 5: — A shortened treatment with TEAAA is sufficient to minimise non-specific probe binding in SDS-treated samples. The background stain in the shell field periphery (identified in Figure 3) is also observed for SDS-treated samples (arrows in A), as represented by a probe against the gene “contig 380566”. Note that after treatment with SDS, the protonephridia are stained (arrow in B). Both non-specific WMISH stains are strongly reduced after one incubation step in TEAAA (D-F) and disappear after two incubation steps (G-I). Larvae in A, D and G are shown from a dorsal perspective, and larvae in B, E and H are viewed from lateral. Panels B, E and H are reflected about the vertical axis for clarity of presentation. [file 12861_2015_68_MOESM5_ESM.pdf]
